# Active Aging Governance and Challenges at the Local Level

**DOI:** 10.3390/geriatrics6030064

**Published:** 2021-06-25

**Authors:** Alexandre Fernandes, Teresa Forte, Gonçalo Santinha, Sara Diogo, Fernando Alves

**Affiliations:** 1 GOVCOPP, Department of Social, Political and Territorial Sciences, University of Aveiro, 3810-193 Aveiro, Portugal; alexandrefernandes@ua.pt (A.F.); teresaforte@ua.pt (T.F.); 2 CIPES, Research Center on Higher Education Studies, 4450-227 Matosinhos, Portugal; sara.diogo@ua.pt; 3 CITTA, Department of Civil Engineering, University of Porto, 4200-465 Porto, Portugal

**Keywords:** active aging, local policies, governance, stakeholders, older people healthy life

## Abstract

International and national guidelines have been promoting active aging while creating the necessary means for decision-makers and other relevant actors to work together (governance mechanisms) to implement local and active aging policies. This is especially important in the present COVID-19 pandemic context, posing greater challenges on older people who tend to be self-isolated. How are local actors conceptualizing active aging? What are their priorities related to a healthy life for older people? Which governance mechanisms are used to implement such policies? These are some of the questions addressed in this paper, targeting Portugal, a southern European country. A mixed-methods sequential explanatory design combining a survey conducted at a national level and interviews with key actors in the NUTS III Aveiro Region was employed to identify and understand the underpinning governance mechanisms. Findings confirm the ‘passive organization type’ in which European politico-territorial studies tend to place Portugal, as there are gaps in the way policies are formulated, implemented and evaluated, as well as a lack of coordination. Results of this study have important impacts on the way local governments and other stakeholders will prepare themselves in the post-pandemic period to design and implement policies addressing active aging.

## 1. Introduction

Populations in developed countries are aging rapidly, and an increased number of older people is expected to live longer [1,2,3,4]. Amongst the European Union member-states, Portugal is already the third-most aged country, in which approximately 21.5% of the population is more than 65 years old [5,6]. Although this phenomenon results in several positive societal factors, it also creates countless individual and collective challenges. Up until the 2000s, debates and policies on population aging envisaged mainly the physical and psychological deterioration of older people along with the need to provide services capable of coping with such limitations. Since then, a new representation of older people has gradually emerged [7,8,9], mainly influenced by two aspects. Firstly, aging is a market segment of millions of people. A new image of older people—appearing as active, consumers and participating community members—captured the attention of both marketers and decision-makers. Secondly, expectations towards public policies increased as older people became more demanding, mostly due to the growth of literacy rates visible among all Organization for Economic Co-operation and Development (OECD) countries. Accordingly, seniors began to reflect and even to fight for their economic, social and cultural rights in order to be an active voice in decisions concerning their lifestyles [10,11,12].

More efficient response to the aging phenomenon relies on a multi-scale intervention at a public-policy level, involving various public entities at local, regional and central levels—a whole-of-government approach—in close collaboration with other stakeholders whole-of-society approach. The challenges posed by this cooperation should be considered by policymakers in the design and implementation of policies promoting active aging and catalyzing efforts in order to improve the physical and social environments for seniors [13,14,15]. In fact, political decentralization has been supported on the grounds of (more) responsiveness to local needs and differences in political demands, fostering, in this way, political variation and innovation [16,17].

Successful policy design involves network governance in which government action interlinks first with decentralized institutions and, more recently, with the private sector and civil society actors in order to aggregate the real needs of its users. According to Enroth (2014) [18], governance as networks became the privileged term in an ideal-typical trichotomy of forms of governing—hierarchies, markets, and networks—which allowed for the perception of a step-wise evolution over time. Such emphasis on networks underlines the need for collaboration to get rid of silos, compartmentalization, and polarization [19].

In policy design, it is important to distinguish between “condition” and “problem”. It is generally accepted that a person’s lifestyle adapts as one gets older and conditions change [20,21]. However, according to Graycar (2018) [22], when conditions assume the form of economic deprivation, health complications and/or dependency, these turn into problems, drawing attention to policy design processes [23,24]. In fact, planning for health is one of the growing concerns of the last decades [20,21,25,26]. By reinforcing the active participation of older people [27], the new governance paradigm requires the need to shift public policies and competencies so that multi actor and multi-level governance can operate in networked cooperation. In particular, it is argued that networks may well be the most suitable governance mode to tackle these so-called wicked problems, including active aging, which must be addressed by bringing together resources of different organizations and interest groups [28].

The World Health Organization (WHO), the Organization for Economic Co-operation and Development (OECD), and the European Commission (EC) concomitantly refer to active aging as a process not only of the individual but also of collective responsibility, implying productive activities, being them economic or social, without putting aside the physical and mental well-being [29]. Active aging can be measured at an individual level, unifying the components promoted by policymakers, researchers, and the elderly’s own perspectives on active aging [30].

Accordingly, national and European Union policies seek to overcome social imbalances resulting from an aging society, such as the pension system, social security, and restrictive measures harmful to the interests of retired citizens [31]. Being a crucial element in the central government/society dialogue [32], local governments and local stakeholders must act as key players in fostering active aging. According to the Age Platform Europe (2011) [33], they are in the best position to meet the older population’s needs and challenges, as they are at the forefront in capitalizing on active aging opportunities.

The design and implementation of aging policies aiming at improving the living conditions of the population depend on the involvement of several actors, either public or private, such as local governments, non-profit organizations, among others [34]. The studies developed by Barbosa (2015) [35] and Bárrios (2017) [36] for Portugal conclude that local governments, endowed with skills and resources, play a meaningful role in improving the conditions of older people due to their greater proximity to citizens and the capacity to mobilize the remaining local stakeholders. The role of such stakeholders and local governments in promoting and implementing active aging policies and initiatives gains additional significance in the present pandemic context, as the sense of balance previously achieved between age-related syndromes and good quality of life and good health is now under higher pressure [37].

With a share of older people representing a considerable part of the population, public policies in Portugal have been devoting particular attention to this aging phenomenon, materialized in 2017 with the publication of the Active and Health National Strategy 2017-25. Research on risk factors, clinical situations, and social and economic status point out the main reasons: Portuguese older adults are a vulnerable group in terms of poor socioeconomic conditions and unhealthy lifestyle behaviors, with multiple chronic health problems, and many can be classified as inactive and with difficulties in communication with others [38,39]. Still, to the best of our knowledge, there is a lack of studies focused on how Portuguese local governments and other local stakeholders approach the phenomena of active ageing. This study, of an exploratory nature, contributes to this topic guided by the following research question: “How is active ageing understood and put in practice at a local level?” and corresponding objectives:

− To explore what features the representation of active ageing policies held by different local stakeholders;
− To detect the priorities and challenges of the process of local policy definition and implementation;
− To identify the positioning and reach of local actors in the development and implementation of active ageing policies, the horizontal cooperation between the envisaged entities and wider networks;
− To perceive the governance mechanisms involved in the process of policy design and implementation of active ageing and how they interact at different scales (local, national and European).

The remainder of this paper is structured as follows. The next section presents the methodology employed to understand how local governments and other local stakeholders perceive aging and which policies and/or initiatives they prioritize. Section three presents the study findings, being followed by the discussion, interpreting its results and policy implications, concluding with the limitations and the conclusions, setting the stage for a wider discourse on local aging policies.

## 2. Materials and Methods

A mixed-methods sequential explanatory design with two consecutive phases was adopted. Accordingly, the study ranges in focus from general perceptions about active aging and knowledge on related public policies, collected in a first phase through a questionnaire, to more detailed insights from key actors, obtained through semi-structured interviews in a second phase. The first phase was conducted at the national level. The case selection for the second phase—aimed at identifying specific networking dynamics, not accessible otherwise—explored what was typical and common within the same territory, more specifically NUTS II Centro Region. In particular, the choice of this region of NUTS III Aveiro Region (Aveiro is the second-most populous city in the Centro Region of Portugal) was due to a higher familiarity of the authors with this specific context allowed more anchored interpretations of the findings.

The questionnaire applied in the first phase focused on: (i) the identification of active aging-related concepts and respective source (ii) the priorities and strategies to be adopted by the Local Council tackling challenges posed by an aging population; (iii) the identification of implemented policies, cooperating entities and main outcomes. The sample included key actors working in institutions selected for their potential to promote active aging: Local Governments (LG); Senior Universities (SU); charity institutions of “Santa Casa da Misericórdia” [Holy House of Mercy] (SCM) (most with more than 500 years and with the mission to support the sick, the disabled and the older people) and Community Care Units (CCU) (which provide healthcare, psychological and social support at home and community level), covering the entire national territory. These institutions have a relevant role in the Portuguese context regarding the social, education and health domains addressing the elderly population, being privileged areas to apply the concept/object of study. The individuals within these organizations who were invited to participated ranged from actors holding top and middle management positions (e.g., rectors; directors of the institution and/or department) to more technical roles (e.g., social workers; line managers).

From December 2017 to June 2018, the questionnaire was sent via LimeSurvey to the entities mentioned above, having an average response time of 10 min. From the 482 answers collected, only 153 (31.7%) were completely answered, hence considered valid, retained and analyzed: Local Governments (*n* = 96 of 308 ≈ 31%); Community Care Units (*n* = 24 of 262 ≈ 9%); “Santa Casa da Misericordia” (*n* = 23 of 387 ≈ 6%) and Senior Universities (*n* = 10 of 305 ≈ 3%). The reason why there is such a disparate value between the total responses collected and those completely validated is due to the fact that as soon as the inquired person clicks on the questionnaire—just to check and/or open the link to access the questionnaire– it was counted as an input value (questionnaire).

After finishing the first phase of data collection, the institutions from Aveiro Region that agreed to participate before were contacted and asked to collaborate in the second phase of data gathering through semi-structured interviews. From a total of 11 Local Governments surveyed, four agreed to participate. From the 11 “Santa Casa da Misericórdia”, 11 Community Care Units and 11 Senior Universities surveyed, only one of each agreed to collaborate in this second phase.

Aimed at exploring in depth (i) the process of design and implementation of active aging-related policies at a local level and (ii) the nature of the interaction and networking established between local, regional, and national entities, an effort was hence made to include at least one representative from each institution surveyed. A sub-sample of 7 institutions agreed to collaborate in the second phase—LG (*n* = 4); SU (*n* = 1); SCM (*n* = 1) and CCU (*n* = 1). The Local Governments interviewees were city councilors and the Senior Universities, “Santa Casa da Misericórdia” and Community Care Units were directors/coordinators of these institutions.

The interview script is aligned with the aforementioned objectives. To explore the first objective related to the representations held about active ageing initiatives and policies, the following questions were put forth: “What do you understand by active ageing?”; “What role should older people have in local politics?”

To address Objective 2 and 3, unveiling the main priorities and challenges in the development and implementation of active ageing policies as well as to the positioning and reach of local actors in this process, the following questions were asked: “Which policies are being privileged?”; “Which competencies should your organization have to address active ageing and which are the challenges of an aged municipality?”; “What are the short/medium and long term strategies to implement active ageing?”; “How does the process of policy design for active ageing unfolds? (which actors, who participates, funding, according to which evidence and means)”.

Finally, drawing on a multilevel perspective, and in order to address objective 4, unveiling the interconnection between policies defined at a local level and other government levels, namely regional, central and European, the following questions were put forth: “Which policies were designed according to national and European guidelines?”; “Is there intermunicipal collaboration in designing ageing policies?” “What should be the role of local and central power in the active ageing policy design?”

The script for Local Governments institutions and the other three organizations—Senior Universities, “Santa Casa da Misericórdia” and Community Care Units—differ slightly, given our goal of understanding the governance mechanisms between institutions (local, regional, national and European).

The interviews were subjected to a thematic analysis. The deductive coding scheme of themes was outlined in advance for each question according to the relevant literature, and the responses were analyzed by two independent coders using NVivo 12 software. The unit of analysis was the complete response to each question from each participant with a dichotomous coding, i.e., either the theme was present or not and no content within the same response was coded twice with the same theme. The theme’s presence allows a comparison between subjects’ perceptions and viewpoints as well as an understanding of how disseminated it is across different institutions. The frequencies of themes per question are organized from the highest to the lowest to better grasp its salience in discourse. This hierarchy is a proxy to the importance attributed to certain themes within the subject.

## 3. Results

### 3.1. Facts and Figures—The Relevance of Concepts, Priorities and Strategies

The first phase of data collection was based on the questionnaire, which, as aforementioned, comprised three main sections: (i) identification of the most relevant active aging-related concepts and respective sources of knowledge. A set of concepts and possible sources were included in the questionnaire and the participants had to indicate which they were aware of and, for each affirmative reply, how they came to know them; (ii) an appraisal, through a Likert scale (1—highest priority and 5—lowest priority), of the priorities and strategies to be adopted by the entities at stake to tackle the challenges posed by an aging population; and (iii) identification of implemented policies, cooperating entities and main outcomes. Bearing these objectives in mind, sociodemographic data of respondents, e.g., age, gender, disciplinary field/area of training, and years of service, were not requested as it was not intended to analyze the respondents’ perceptions and attitudes as decision-makers/experts, but rather to collect information by local organization and type of actor. Accordingly, respondents were asked to indicate only the role/service they perform at their institution (Table 1).

The analysis of the questionnaires allowed us to identify the presence and prevalence of patterns (themes) in the discourses of the different institutions’ representatives regarding two main axes of analysis: (i) the process of elaborating and implementing active aging policies at the local level; and (ii) the way(s) local actors interact with other local, regional and national entities (i.e., governance mechanisms). These ideas will be deeper elaborated on later in this study.

Table 2 shows how familiar the respondents are with the concept of active ageing and similar expressions usually used in literature—healthy ageing, productive ageing, healthy cities, age-friendly cities.

With respect to active aging and healthy aging, more than 90% (*n* = 148) of respondents were familiar with both “active aging” and “healthy aging” expressions. These are, in fact, the most widespread concepts, especially in the context of local governments with a rate of 99% (*n* = 95).

On the other hand, the concept of “productive aging” is not well known and in SCM and CCU, only half of the respondents were aware of their use. A possible explanation for this is that most of them do not hold any leadership position or responsibility for the organization, and therefore do not need or feel the need to be updated at that level. The fact that the participants of the local governments are more familiar with this concept suggests that knowledge is contingent/tangled to the nature of the domain in which these entities operate. In fact, both SCM and CCU mostly deal with older people with various fragilities, especially health-related, which are not consistent with this representation of aging [productive; active], while LG and SU are at the forefront in promoting more active role for older people. Declining physical and mental health, loss of functional capabilities, and weakening of family and social ties represent a significant barrier to active aging in a context of institutionalization [40].

A similar pattern is found in the results associated with the concepts of “healthy cities” and “aged-friendly cities”. These are more widespread in LG and SU than in SCM and CCU, which is understandable considering that the design and implementation of such initiatives are a municipalities’ responsibility. In fact, the analysis of the sources of these concepts’ knowledge corroborates the trend described above. In local governments, the concepts derive from the institution’s own orientation or from European orientation and/or guidelines, which can be explained, namely regarding the first option, by the fact that most respondents are senior technicians (graduated staff); this implies that they receive information by standards and regulations issued by the entity where they work and to whom they are accountable to. In the case of SCM, the results are dispersed, but the concept of “productive aging” is also highlighted in order to draw our attention to the lack of knowledge above emphasized. In this regard, and since most respondents are aware of this concept by their own initiative (i.e., individual choice) and only 10% are conscious of this due to the institution’s guidance, one may argue that this is not a relevant concept for these key actors. Finally, in CCU, it is possible to verify that concepts are mostly obtained by respondents’ own initiative and, only seldom, through the organization’s own guidance, a fact that may reveal that these key actors are not yet aware of these issues. In SU, by contrast, knowledge comes mostly from the institution itself—which clearly complies with the nature and mission of these organizations. In the following sections, a discussion on these representations and subsequent policies and strategies is presented.

With respect to the main strategies these entities adopt to tackle the challenges posed by an aging population, findings show that LG prioritizes the promotion of healthy lifestyles and leisure activities as opposed to the design of public spaces without restriction for pedestrians. This may mean that most municipalities have addressed such concerns and are now one step further looking at health lifestyles. Similar findings can be found in other contexts. In Spain, for instance, Fernández-Ballesteros et al. (2010) refer to regular physical activity and a healthy lifestyle as the most common predictors present in the daily lives of the old people [41]. This position from LG is also in line with the perspectives presented by the SU, which consider that the design of public spaces is not a priority. However, as seniors who attend universities are usually more active and more autonomous, the added value that the old person can present in the decision-making processes is a priority. Unlike LG and SU, SCM and UCC priorities address urban planning concerns, namely the design of public spaces, the issues of public space design without restriction for pedestrians, and the promotion of a good transport network that avoids social isolation. A possible explanation is that the target population of these institutions is more social and physically vulnerable.

Regarding policies, most of them are designed by LG, though the other entities do, in some cases, participate in their development. The largest number of initiatives are in the social action domain, with LG mostly financially supporting other institutions. Other initiatives concern the promotion of health education and sports activities. Local partnerships tend to prevail in these initiatives, mainly through the Social Network, a set of organizations led by the LG that plan and execute public policies in a collaborative environment [42]. The second phase of data gathering would complement and enrich these findings.

### 3.2. Representations about Active Aging Initiatives and Policies

The seven interviewees’ (henceforth referred to using the acronyms of the respective institution) conducted in the second phase suggest a multilayered representation of active aging (see Table 3).

The deductive thematic analysis of the answers to the question “What do you understand by active ageing?”, using categories informed by the literature on aging and active aging, indicates that Cognitive and Psychological processes (*n* = 15) and Health and Well-being (*n* = 13) are the most salient dimensions of the representation. The first is evidenced in the responses of five participants alluding to the training and maintenance of cognitive skills (e.g., memory, learning, problem-solving) as well as emotional regulation to cope with typical adversities (e.g., loss of capacities, autonomy, isolation). Being the most salient dimension for LG3, SU, and SCM, it is important to highlight that LG3 and SU consider it from a positive viewpoint, while for SCM, the focus is on preventing and repairing losses, which also aligns with the specific features of the population they deal with.

*“The ability of the individual to adapt to the ageing process, self-regulate, make decisions and choices” *

*(LG1)* 

*“I prefer to refer to as lifelong learning” *

*(LG4)* 

Health and Well-being, albeit coming second in terms of references, is mentioned by more participants (*n* = 6) either directly or through more general allusions to Healthy Lifestyles and Quality of Life. Interestingly, Senior Universities was the only participant that did not address it, rather focusing on Cognitive/Psychological Processes (*n* = 3); Cultural Factors (*n* = 2) and Sports/Physical Activity (*n* = 1), which is probably fine-tuned with the population they mostly contact with.

*“The process of maximizing health opportunities” *

*(LG1)* 

*“Ageing in a healthy way” *

*(LG3)* 

Participation in society is the most important dimension for LG1 (*n* = 5) and CCU (*n* = 2) and the second more important for LG2 (*n* = 2), swiftly followed by Sports/Physical Activity that is mentioned by LG1 (*n* = 2), LG4 (*n* = 1) and US (*n* = 1). Leisure and Occupation (LG2, *n* = 3; LG4, *n* = 2) as well as Cultural Factors (LG1, *n* = 4 and US, *n* = 2), conveying a broad understanding of social and cultural forces that model and influence how older people are perceived and valued by society at large.

*“The saying “to stop is to die” reinforces that we should always be moving, which requires quality in the ageing process” *

*(US)* 

*“To participate and enjoy of what the community, municipality and all the structures and organizations have to offer “*

*(LG2)* 

When asked about the role that older people should have in local politics, Participation and Decision-Making is consensual with 11 mentions, followed by Volunteer activities (*n* = 2) and Promotion of Culture (*n* = 1).

This suggests that the conceptual representation is coherent with positive attitudes and behaviors towards a higher engagement of older people in local politics and policy-making.

*“We have seniors with high academic degrees and try to benefit from the wisdom they may convey. This “Senior Assembly” is a way to include older people in local policy. The same is true for our “White Hair Festival”, entirely designed according to the preferences and wishes of this age group” *

*(LG3)* 

*“We should have a kind of council of older and wise” *

*(LG4)* 

In sum, all of the interviewees show a growing concern with older people having an active voice within the community. For local government individuals, one of the privileged venues has been to promote debates and discussion forums that allow them to engage and partake in at least the initial phases of the policy-making process. In fact, according to the Active Aging Index (2012), similarly to Estonia and Romania, Portugal is one of the three countries with the lowest rate in this domain [43].

### 3.3. Local Governments Competences, Intervention, and Strategy

Comparing the main competencies and challenges of Local Governments for Active Aging—identified by the seven participants—with what they outlined as current areas of intervention in age-related policies, one can assert that, in general, Local Governments’ areas of intervention are tuned with the main challenges and Local Governments competences on the matter.

LG1, which highlights *Promotion of Health and Well-being* and *Raise Awareness on Benefits of Sports/Physical Activity* as main competencies, has *Sports/Physical Activity* as the main area of intervention (“Sport for all” and “Sport is life” are local mottos) swiftly followed by *Leisure and Occupation Activities* and *Foster Participation*. The latter two, in turn, are coherent with the competence and challenge of *Integration in Society* and *Inclusion in Local Decision-Making*.

*“Inform the population of all the benefits entailed by the regular practice of physical activity and sports” *

*(LG1)* 

*“It is important to value their knowledge and experiences, including them in local decision-making” *

*(LG1)* 

In a different vein, LG2 puts forth the competencies of Local Governments in Offering Leisure and Occupation Activities and Social Support/Benefits, which matches the emphasis given in practice to Fight Isolation and Loneliness and Security.

*“Foster leisure activities, games and ways of occupying time for retired citizens” (…) we offer a senior card with discounts “*

*(LG2)* 

A less obvious connection is found in LG3 and LG4 hierarchies. LG3 emphasizes the need to allocate Physical and Human Resources to fight an aged municipality and indicates only one area of intervention, namely Fight Isolation and Loneliness. To Fight Isolation and Loneliness is, in turn, referred to as the most important challenge and LG competence by LG4 alongside Social Support/Benefits. The competencies and challenges carry a tone of reparation and protection, contrasting with the more proactive current areas/aims of intervention Promotion of Health and Well-being, Leisure and Occupation Activities and Sports/Physical Activity. Engaging older people in leisure activities and healthy habits can indeed have a positive impact on isolation and loneliness, granted they adhere to the initiatives.

*“The municipality has to possess the resources to implement strategies to improve aging processes” *

*(LG3)* 

*“The great challenge is to develop activities and policies adaptable to a heterogeneous aging population” *

*(LG4)* 

One result worth pondering is the low frequency of mentions to urban planning, physical space and accessibilities either in Local Governments competencies/challenges as in Local Governments areas of intervention. As shown in Table 3, it only appears twice (LG1 and LG2) with low salience.

Perceived Competences and Challenges and Main Areas/Aims of Intervention (Table 3) triangulate with Future Strategies for Aging. Curiously, Accessibilities are heavily emphasized as part of the strategy of LG1 for Aging alongside Health and Well-being, Sport/Physical Activity, Leisure and Occupation and Support/Benefits. LG2 highlights Inter-generational support and the need to conduct periodic studies/surveys to better grasp ongoing challenges and necessities. These studies are the only strategy mentioned by LG3. Finally, LG4, besides also endorsing the periodic studies, prioritizes Supports/Benefits, Fight Isolation and Loneliness; Sport/Physical Activity and Leisure and Occupation. The consensual view on regular studies is a positive aspect to be highlighted.

When asked about the share of the budget allocated to current and forthcoming age-related policies, LG1 mentions that the funding is approximately 65,000 euros and it is self-generated; LG2 indicates 10,000 euros allocated to the indirect support of many activities carried out by different actors who benefit from Local Government programs; LG4 indicated a precise value (62,000 euros), corresponding to a specific domain “Pelouro Maior Idade” (aging office) included in the Social Action Services for Inclusion and Citizenship; and, finally, LG3 only refers that it carries costs with infrastructures and HR, which was too disperse to aggregate it in a specific value.

### 3.4. Design and Implementation of Active Aging Policies: Diagnosis, Horizontal Cooperation and Governance Mechanisms

The diagnosis of issues requiring intervention, according to LG1 and LG2, converge on three main venues: Social Network; Local Council of Social Action (CLAS) and specific diagnostic studies, often conducted by these two entities with a collaborative mindset. LG1 also includes informal surveys to the community and sets forth the:

*“Social Charter for Older People elaborated and made public in 2018 as an information, characterization and analysis tool is an auxiliary document for the process of connection and adequacy of social policies, planning of supported associations actions and decision-making support.”*

LG3 and LG4, similarly, endorse the periodic studies coined as “social diagnostics”.

Even though we lack specific details concerning the process, the issues at stake are usually identified through specific studies, data gathering with the community and meetings with the institutions from Social Network and Local Council of Social Action (platform of debate, at local level, focused on the participation, representation and collaboration between public organizations and private social initiatives). The work developed by these organizations is viewed by our interviewees as crucial for promoting active aging practices, particularly due to their ability to motivate local entities to work together for a joint cause. Intermunicipal Communities and Supra-Council Platforms (SCP). SCP, a member of the Social Network, is also perceived in a positive way, given their role in optimizing the planning and management of financial and social resources.

Consulting multiple actors and fostering of co-responsibility is to be perceived as a very positive sign. Crossing Local Governments responses with the other surveyed institutions, it is observable that Local Governments cast a relatively wide net of viewpoints on age-related topics, which includes studies from in-house and associations—Social Network and Local Council of Social Action—official statistics, surveys and other original empirical research and evidence from interested stakeholders. The implementation itself does not follow a standard procedure, ranging from individual initiatives to group-based processes mediated by local power through Social Network and Local Council of Social Action.

When asked to describe the process of designing active aging policies, particularly with respect to actors, context, participants and funding, the interviewees were able to summarize this complex interaction in a few main vectors depicted in Table 4. 

As it was possible to verify, all the institutions privilege a networking approach gravitating towards the common ground of Active and Healthy aging with a participatory and inclusive approach.

This networking is allied with another specific feature highlighted by our Local Governments participants and corroborated by the institutions’ partners: “Self-funding and support of institutions”. Regarding the other surveyed institutions, the common denominator is that, albeit, with the scarce funding from Local Governments, they absolutely depend on them as well as on partnerships established between local power and IPSSs (non-profit institutions).

The funding of initiatives and projects may have different sources: (i) included in the annual budget and allocated to a specific Local Governments domain; (ii) deriving from applications to projects’ grants at national and international level and (iii) resulting from fundraising campaigns, a reality very common in Anglo-Saxon settings, and that is growing in the Portuguese context.

It is worth mentioning that volunteering activities and projects enable the financial feasibility of these initiatives, as well as the deepening of intergenerational bonds within the community. Here, the aforementioned work of the Social Network is essential at different levels, rendering an inter-institutional partnership according to the new generation of active social policies.

All Local Government actors agree with the importance of following national and European guidelines on the matter, emphasizing the funding opportunities that help them to develop specific policies. For instance, LG1 states that *“All of our policies anchor on European and National directives and guidelines (…) usually we are able to anticipate the issues with Social Network filter and our partners’ contributions.”*.

Similarly, SCM and SU try to be updated and aware of national/European guidelines and initiatives. Moreover, some of the past programs were conducted accordingly and there are human resources allocated to search and apply to this funding. The CCU, on the other hand, has no specific national and/or European program applied to active aging, except for initiatives commemorating special days (e.g., “National Day of”; “World day of”).

More studies, guidelines, funding, policies and agendas are the type of responsibilities that the participants attribute to the Central Government. The local level is perceived as quintessential given their extensive knowledge about local realities but it is the Central Government’s role to foster these interventions.

This idea is endorsed by the Local Government actors as well, who agree in recognizing the importance of local power that does a lot without sufficient resources.

*“The promotion of older people rights, assistance and participation should be defined and framed by the Estate, but is the local power that structures and adapts them to local needs.” *

*LG2* 

*“The Central Power provides general guidelines, locally adapted to the specificities of each place.” *

*LG1* 

*“There is no doubt that the responsibility should be of Local Power, but Central Power must capacitate them financially.” *

*LG3* 

*“If there is no responsibility of Central Power, Local Power must come forth (…) The responsibility is not necessarily ours, the municipality funds initiatives out of choice (…) *

*LG4* 

Over the years, they had to adopt clever networking-based solutions to advance with their initiatives, to which Intermunicipal Communities and SCP were fundamental as intermediaries with central government. The criticism to central government involvement, engagement and accountability on age-related matters is both manifest and latent. All agree that the Central Government should emit more guidelines and offer more support, financial and otherwise. This is even more true considering the wide impact of an aging population that is not circumscribed to regional and local spheres. Accordingly, one of the most salient problems identified is the lack of efficient multi-level cooperation. This is the case of the relation between local government and intermediary and central instances, especially regarding a clear definition of roles and inherent responsibilities. Without specific guidelines on the matter of aging provided by the central power, there is a disparity between regions, given that most of the initiatives rely on local contextual features, such as demographic and financial aspects.

According to Matos (2013) [44], the improvement of multi-level cooperation may result from connecting Local Council of Social Action and Intermunicipal Communities and Supra-Council Platforms (NUT III), at a council level, to the Social Security Institute, at a national level, which, in turn, could respond directly to the central power. This model enables the cooperation of different levels of power, either between actors as well as policies and instruments. The interviewees mention this cooperation, agreeing on the relevant work conducted by the Local Council of Social Action and SC. However, the connection between Intermunicipal Communities and SCP and central government is still perceived as inefficient.

It should be highlighted that the interviews were conducted in Aveiro Intermunicipal Community which may not be representative of the prevailing culture of participation about age issues in Portugal. In recent years, this region has been particularly prolific in tackling some of the physical and social constraints related to this age group. What is more, a concern was noticeable with consulting the population in order to identify existing problems and define measures to address the specific needs of citizens’.

## 4. Discussion

The design and implementation of active aging-related policies require a broader view of the multidimensional nature of the aging process. True, political agents are increasingly thinking and debating the pervasive issues of population aging, and this discussion gains renewed attention towards the COVID-19 pandemic [45]. However, as argued by Walker and Maltby (2012) [29], even though active aging is widely promoted by the (discourses of the) EC, this does not automatically translate into devising and designing policies to be implemented. This challenge is even greater when one narrows the level of implementation, as this study evidenced. The literature tends to blame policymakers and social policy analysts for neglecting aging [29,36,46], though, at present, it is officially acknowledged as a “major challenge”.

The results of our study shed some light on the strengths and shortcomings of such a challenge as it is perceived by the key actors. As Díaz Orueta (2006) [47] puts it, governance, through the actions and choices of local authorities, is key to successfully respond to today’s complexity of the local government, whose decisions and policies affect individuals’ sense of belonging to a democratic society.

In terms of policy design and implementation, one of the most salient issues is the lack of clear cooperation between levels of power, which is of paramount importance to approach some of the challenges posed by an aging population. Collaboration is also essential for a successful implementation of planned actions and procedures in the area of public health emergency response, even in non-European settings [48]. This is also a key element put forward by Ferrão (2015) [49], who posits the importance (as well as the challenge) of multilevel governance to public policy design and implementation while arguing that it can only be efficient as much as the needs, priorities and capacities of private and public actors and/or agencies are all considered. This is aligned with the view on the importance of consulting the local population on a regular basis to have an informed insight into specific concerns, needs and limitations. This public consultation is also significant to identify existing problems and define measures able to address citizens’ specific needs—again, a paramount concern, especially valuable in the present pandemic time. In turn, these perceptions corroborate, or reinforce, the results of this study, showing that the concepts of “healthy cities” and “aged-friendly cities” are the best known by Local Government actors, who will employ the support of local actors, volunteering activities and participation of individuals as vehicles stimulating the process of age-related policy-making. These results are in line with Flores, Caballer and Alárcon (2019) [50], Principi et al. (2013) [51], and Principi, Chiatti and Lamura (2012) [52] findings.

This leads us to the paramount role of networking for more efficient governance mechanisms, namely, the design and implementation of active aging policies and initiatives. As aging is a concept that encompasses many areas, local governance mechanisms play a crucial role herein by enabling a network of partnerships between local entities, ranging from third sector institutions, public and private providers, academic institutions and civil society. In this regard, the Social Network is a strong lever to aggregate and get the local agents in constant cooperation. However, the absence of a multilevel view (encompassing the central level) limits the scope of the initiatives, which are mainly focused on the promotion of healthy lifestyles and leisure and occupation activities, as there are no guidelines from the central government that may serve as a guide to local government. As for the intermediate structures, namely at the level of the Intermunicipal Communities, the interviewees identified the need for these structures to consolidate their role as places for debate and sharing, for policy implementation, for clarification and, above all, for monitoring what is at stake.

## 5. Conclusions

The results obtained with this study allowed a deeper understanding of what is being outlined at the local level regarding active aging, while identifying some of the inherent mechanisms of the decision-making policies process. Firstly, it was possible to identify the areas in which organizations were most involved, namely the promotion of healthy lifestyles and occupational and leisure initiatives, with a very strong commitment on the part of local governments in areas of social security and financial benefits. Secondly, it showed the prominence of a culture of decision-making in networks, based mostly in the Social Network. Finally, it corroborated some concerns expressed by research on governance within the Portuguese context, specifically regarding the lack of guidance from the central administration on these matters, as well as the low interconnection between the different governance levels. In this context, it was possible to observe that an intermediate level of governance may be the key to these issues, with the Intermunicipal Communities and Supra-Council Platforms holding the potential to foster debate and common practices among the various municipalities in a region.

## 6. Limitations

Two main limitations of this study should be mentioned that may be tackled in further research. The first refers to the size of the sample, which was relatively small, hence not allowing a generalization of the conclusions. In fact, the outcomes of this study refer to a very specific geographical setting, molded by its own historical, cultural and political-administrative specifics, and thereby more research is needed before generalizations or extrapolations can be elaborated. Notwithstanding, it would be of great value to compare the differences and similarities between the Portuguese context and other European countries, especially in what concerns the way different countries elaborate their policies considering European guidelines.

Bearing in mind the actual moment of questioning and reflection on the older people brought by the COVID-19 pandemic, this research provides interesting results for policymakers and is worth consideration in addition to other international and national contexts. This brings us to the second limitation (and potentiality) of this study: the importance to combine our approach with a mapping of these policies in the Portuguese territory to understand the different geographical contexts (coastal/inland or rural/urban), and the extension of the study to other regions of the Portuguese territory to explore if these findings are context-dependent or reflect a more national-based reality.

## Figures and Tables

**Table 1:** Role/service they perform at their institution.

Type of Institution	Sample	Respondents’ Profile
Local Government	*n* = 96	Senior technician (*n* = 52) Head of division (*n* = 26) Executive Board (*n* = 18)
Santa Casa da Misericórdia	*n* = 23	Technical director (*n* = 9) Senior technician (*n* = 6) Head of division (*n* = 4) Ombudsman (*n* = 3)
Community Care Units	*n* = 24	Coordinator (*n* = 13) Nurse (*n* = 10) Physiotherapist (*n* = 1)
Senior Universities	*n* = 10	Director (*n* = 5) President (*n* = 2) Teacher (*n* = 1)

**Table 2:** Knowledge of the actors for some key concepts of aging.

Type of Institution	Active Aging/ Healthy Aging	Productive Aging	Healthy Cities/Age-Friendly Cities
Local Government (*n* = 96)	99% (*n* = 95)	63% (*n* = 60)	89% (*n* = 85)
Santa Casa da Misericórdia (*n* = 23)	91% (*n* = 21)	43% (*n* = 10)	57% (*n* = 13)
Community Care Units (*n* = 24)	96% (*n* = 23)	50% (*n* = 12)	67% (*n* = 15)
Senior Universities (*n* = 10)	90% (*n* = 9)	70% (*n* = 7)	80% (*n* = 8)

**Table 3:** Dimensions of Active Ageing.

Institution	Themes|Frequency of Mentions
**LG1**	Participation in society|5
	Cultural factors|4
	Health and Well-being|3
	Cognitive/psychological processes|3
	Decision-making processes|2
	Sports/Physical activity|2
	Security|1
**LG2**	Leisure and occupation|3
	Participation in society|2
	Health and Well-being|1
**LG3**	Health and Well-being|2
**LG4**	Cognitive/psychological processes|4
	Leisure and occupation|2
	Sports/Physical activity|1
	Health and Well-being|1
**CCU**	Participation in society|2
	Decision-making processes|2
	Health and Well-being|2
**US**	Cognitive/psychological processes|3
	Cultural factors|2
	Sports/Physical activity|1
**SCM**	Cognitive/psychological processes|5
	Interpersonal relations|4
	Health and Well-being|4

**Table 4:** Main vectors underpinning the process of age-related policymaking.

Institution	Theme|Frequency of Mentions
**LG1**	Participation and decision processes|2
	Self-funding- Support of local actors|1
	Networking|1
**LG2**	Networking|1
	Self-funding- Support of local actors|1
	Diagnose Studies|1
	Centralized policies|1
	CLAS|1
**LG3**	Networking|1
	Assessment of activity|1
	Self-funding- support of local actors|1
	Project grants applications|1
**LG4**	Networking|2
	Volunteering|2
	“Social network”|2
	Activity Assessment|1
	Self-funding- Support of local actors|1

## Data Availability

The data presented in this study are available on request from the corresponding author.
